# Neurosarcoidosis Presenting With Diffuse Multinodular Pachymeningeal Enhancement in the Cervicothoracic Spine: A Case Report and Review of the Literature

**DOI:** 10.7759/cureus.73241

**Published:** 2024-11-07

**Authors:** Amy X Zheng, Ryan G Chiu, Sharon A Secola, Russell Payne

**Affiliations:** 1 Internal Medicine, Methodist Health System, Dallas, USA; 2 Neurosurgery, University of Texas Southwestern Medical Center, Dallas, USA; 3 Pathology, Texas Health Resources, Dallas, USA

**Keywords:** extradural, myelopathy, neurosarcoidosis, nodules, spinal cord

## Abstract

Neurosarcoidosis, particularly in the absence of extra-neurologic systemic manifestations of sarcoidosis, is a challenging diagnosis that has a wide array of presentations. Most often presenting with cranial neuropathies, basilar meningitis or pituitary/hypothalamic dysfunction, isolated involvement of the spinal cord without cranial manifestations is exceptionally rare, often involving intramedullary lesions. Here, we present the unique case of a 64-year-old female with atypical neurosarcoidosis presenting with myelopathy from extra-dural nodules without other neurologic or systemic symptoms. Pathology results from the extra-dural nodules were consistent with a neurosarcoidosis diagnosis. This is the first case report in the literature to our knowledge of atypical neurosarcoidosis presenting as isolated intradural extramedullary nodules without cranial or systemic manifestations of sarcoid disease.

## Introduction

Sarcoidosis is a multisystemic non-caseating granulomatous disease sparked by an immunologic response to an unknown antigen, most typically affecting female patients in the third and fourth decade of life [1,2]. It is a rare pathology but can affect up to 60 out of 100,000 individuals in certain regions and latitudes [1]. The heterogeneous and unpredictable clinical course of sarcoidosis renders its recognition and prompt diagnosis challenging [1]. The typical presentation of sarcoidosis involves the canonically described bilateral hilar lymphadenopathy with lung infiltration, multiple other organ systems (e.g. dermatologic, ocular, osseous, hepatic, splenic, cardiac, and nervous tissue) may be involved, even without a pulmonary presentation [1].

When sarcoidosis involves the nervous system, it is termed neurosarcoidosis, which only affects roughly 5% of sarcoidosis patients [1,3,4]. The presentation of neurosarcoidosis, much akin to sarcoidosis as a whole, is also incredibly variable and can result from involvement of the cerebrum, pituitary/hypothalamus, cranial nerves, meninges, neural vasculature, or peripheral nerves [2-4]. Even rarer than neurosarcoidosis is the incidence of isolated neurosarcoidosis without pulmonary/other systemic manifestations.

Here, we present a case of a 64-year-old female who presented with isolated acute-on-chronic myelopathy due to pachymeningeal sarcoidosis.

## Case presentation

The patient had a history of previous cervicothoracic fusion procedures for spondylotic myelopathy, but despite surgical management of her degenerative pathologies, she developed diffuse multi-focal pachymeningeal nodular enhancement extending from the craniocervical junction to the mid-thoracic spine with progressive myelopathy. She underwent a virtually non-diagnostic biopsy of one of these lesions at an outside facility, positive only for *Propionibacterium acnes*, for which she was placed on antibiotics.

**Figure 1:**
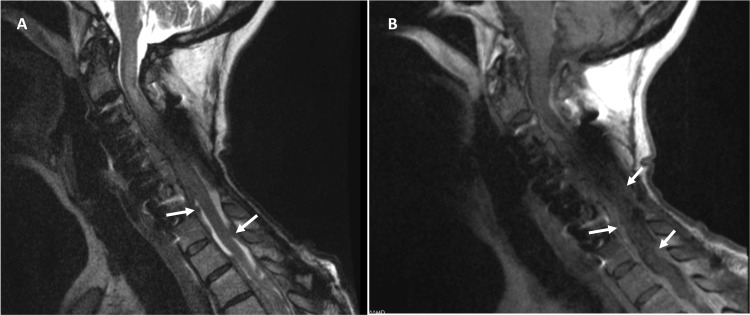
Initial MRI Initial MRI on presentation, with T2-weighted sequence (A) demonstrating cord edema in the upper cervical spinal cord extending from near the cervicomedullary junction inferiorly to roughly the C7 level. Also visualized is myelomalacia distally in the upper thoracic spine likely secondary to circumferential compression from the pachymeningeal pathology. Extramedullary nodular lesions (indicated by arrows) are clearly noted associated with the dura mater. Following contrast administration, these lesions appear to be homogeneously enhancing (B).

She presented to us after a fall with acute onset quadriparesis. Emergent MRI revealed an expansile long-segment T2 hyperintensity, along with enhancing, diffuse pachymeningeal nodularity with cord compression (Figure 1).

Operative management

Due to her acute neurologic decline, she was taken emergently to the operating room. Following the decompression of C2 through C7, the thecal sac was noted to be decompressed. Biopsies of the extradural, dusky-appearing fibrous tissue were sent for intra-operative frozen pathology. This demonstrated atypical large-cell lymphoid infiltrate and lymphocytic predominance. Given this, further surgical decompression was deferred. She was discharged to inpatient rehabilitation after a short postoperative stay and placed on high-dose corticosteroids.

**Figure 2:**
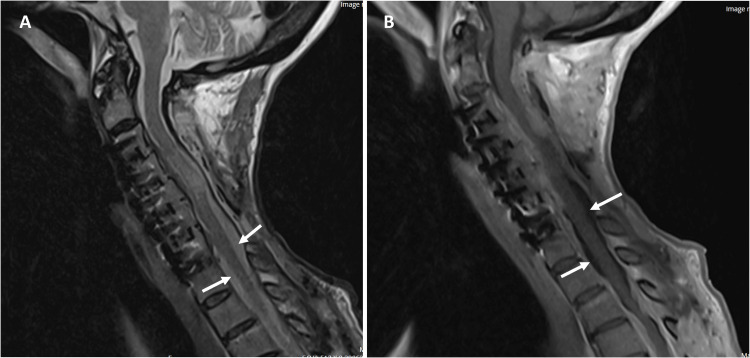
One-month post-operative MRI Follow-up MRI of the cervical spine at one month post-operatively, demonstrating improved mass effect on the spinal cord with improved CSF signal (denoted by arrows) surrounding it, along with diminished contrast-enhancing mass lesion. A T2-weighted sagittal image is depicted (A) as well as a post-contrast T1-weighted image (B).

**Figure 3:**
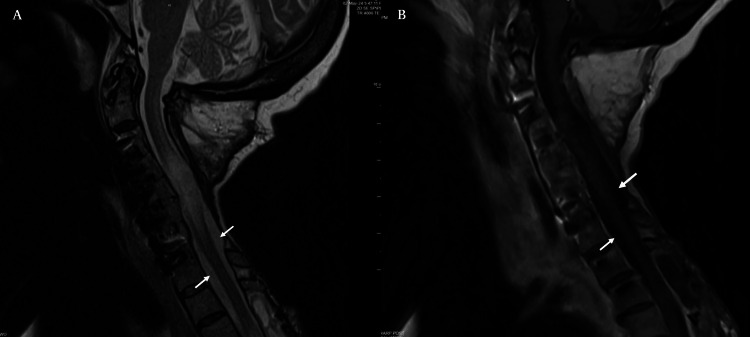
Nine-month post-operative MRI Follow-up MRI of the cervical spine at nine months post-operatively, further demonstrating significant resolution of mass lesions on T2 (A) and post-contrast T1 (B) imaging, with arrows indicating areas of even greater CSF signal, free from prior visualized compressive lesions.

The patient recovered some of her upper and lower extremity strength, began to feed and bathe herself, as well as ambulate using a rolling walker. Short-term (one month, Figure 2) and longer-term (nine months, Figure 3) follow-up MRI imaging demonstrated diminished T2 cord signal and markedly decreased nodular disease burden.

Pathology and laboratory studies

**Figure 4:**
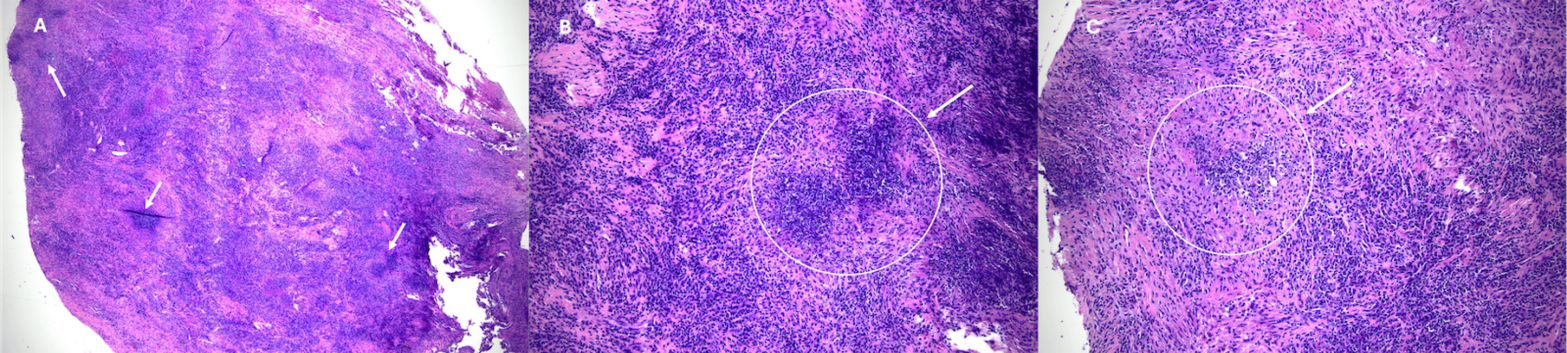
Pathology slides Representative pathology images demonstrating a nodular area of dense mixed inflammatory infiltrate and areas of necrosis (A, 20x magnification), and within this, granulomas with central necrosis (B and C, 100x magnification), which led ultimately to a pathologic diagnosis of sarcoidosis/neurosarcoidosis.

Histologic examination demonstrated lymphocyte-predominant fibroinflammatory tissue, but with occasional granuloma formation, which led to the diagnosis of neurosarcoidosis (Figure 4). These non-caseating granulomatous findings were typical of sarcoidosis and therefore were the main pathology finding leading to this diagnosis.

Corroborating serum studies included elevated serum angiotensin-converting enzyme (ACE) level (91 U/L), correlating with a sarcoidosis diagnosis. Antineutrophil cytoplasmic antibodies (ANCA) testing for vasculitis was negative, as well as AQP4 and MOG antibodies for the workup of neuromyelitis optica (NMO).

Infectious workup was negative, including Grocott methenamine silver (GMS) and acid-fast bacilli (AFB) stains from the specimens, and anaerobic and aerobic bacterial cultures. This also included negative histoplasmosis urinary antigen/serum antibody and negative mycobacterial/tuberculosis labs, VDRL/RPR for syphilis, HIV-1/2 antigen and antibodies, as well as antibodies to *Coccidioides* and *Blastomyces* (given our institution’s location in the southwestern United States), *Aspergillus*, *Brucella*, *Borrelia* *burgdorferi* (Lyme disease), and *Tropheryma* species. IgG4 serum levels were also normal.

CSF analysis was notable for a normal opening pressure, normal glucose, elevated protein (176 mg/dL), and high nucleated cell count (74 cells/μL). No malignant cells were found in the CSF, and like the serum, was also negative for infectious antibodies/cultures. The CSF IgG index was normal and negative for oligoclonal banding.

## Discussion

Neurosarcoidosis occurs in roughly only 5% of patients with sarcoidosis and is unfortunately a known predictor of increased mortality from sarcoidosis [3]. Among neurosarcoidosis patients, it is estimated that 50% to 70% present with neurologic symptoms, with the vast majority of these involving cranial neuropathies. Myelopathy only affects 6-8% of these patients [5,6]. Even rarer is the occurrence of myelopathy without other neurologic manifestations and without any other extra-neurologic systemic systems.

Such is the unique case of our patient, who developed non-spondylotic, progressive myelopathy in the setting of pachymeningeal nodular disease involving the cervicothoracic spine with pathologic findings consistent with lymphocytic infiltration. Here, we will discuss the validity of our diagnosis, as well as compare imaging and pathologic findings with those documented in the literature.

Diagnosis

The current clinical criteria for neurosarcoidosis diagnosis (2018 Neurosarcoidosis Consortium Consensus) is divided into three categories - definite, probable, and possible neurosarcoidosis [5-7]. A diagnosis of definitive neurosarcoidosis requires (1) the correlation of clinical presentation with imaging or other neurodiagnostic studies (MRI, EMG/NCS, etc.) suggestive of neurosarcoidosis after exclusion of other potential diagnoses, in addition to (2) a pathologic diagnosis consistent with neurosarcoidosis. In this case, the patient met both criteria.

In addition to a pathologic sampling of extradural nodules from operative intervention, the patient underwent lumbar puncture and CSF was sent for markers of other autoimmune pathologies, negative for markers of multiple sclerosis and NMO (e.g. no oligoclonal bands) as well as IgG4-related disease, which were all on the differential diagnosis. Instead, elevated ACE levels in both serum and CSF were suggestive of sarcoid.

Furthermore, while it is not presently known, the pathogenesis of sarcoidosis is thought to stem from a reaction to a specific antigen. There is evidence of RNA and DNA from *Mycobacterium* and *Propionibacterium* species isolated from sarcoidosis lesions [8]. This is relevant in this patient with a previous positive culture for the latter.

Imaging

A 2020 series identified four neurosarcoidosis MR imaging-based subtypes: longitudinally extensive transverse myelitis (LETM, most common), short tumefactive myelitis, meningitis/meningoradiculitis, and anterior myelitis [8,9]. Typically, MRI findings in LETM demonstrate a central canal/dorsal subpial pattern of enhancement within the spinal cord itself, rather than the extradural enhancing nodularity with cord compression seen in this particular case [7]. Characteristic imaging findings have also been described as involving nodular and/or linear leptomeningeal enhancement, which is also in contrast to this patient’s findings [6]. Therefore, the presentation of our particular patient may represent a rare, not yet reported MRI-based subtype of neurosarcoidosis.

Pathology

Histopathologically, intraoperative biopsy revealed fibroinflammatory proliferation with lymphocytic predominance with scattered granulomas with negative AFB and fungal staining - a hallmark pathologic finding of sarcoidosis. Additionally, the patient underwent lumbar puncture and CSF was sent for markers of other autoimmune pathologies, negative for markers of multiple sclerosis and NMO (e.g. no oligoclonal bands) as well as IgG4-related disease. Instead, elevated ACE levels in both serum and CSF were suggestive of sarcoid. This lends further support to the diagnosis of definitive neurosarcoidosis.

## Conclusions

This is the first case report in the literature to our knowledge of atypical neurosarcoidosis presenting as isolated intradural extramedullary nodules without cranial or systemic manifestations of sarcoid disease. Neurosarcoidosis should be part of the differential diagnosis for patients presenting with multifocal extra-axial nodularity within the spinal canal. It is also important to be mindful of the wide range of possible presentations of neurosarcoidosis, many of which may be atypical.

At the same time, recognition of current guideline-driven criteria and nuances related to the diagnosis of neurosarcoidosis, in addition to knowledge of its various subtypes, is paramount to effective recognition and, by extension, prompt treatment of affected patients.
